# Radiotherapy and breast cancer: finally, an lncRNA perspective on radiosensitivity and radioresistance

**DOI:** 10.3389/fonc.2024.1437542

**Published:** 2024-09-13

**Authors:** Fatemeh Yazarlou, Ivan Martinez, Leonard Lipovich

**Affiliations:** ^1^ Center for Childhood Cancer, Abigail Wexner Research Institute at Nationwide Children’s Hospital, Columbus, OH, United States; ^2^ Department of Microbiology, Immunology and Cell Biology, West Virginia University, Morgantown, WV, United States; ^3^ Department of Biology, College of Science, Mathematics, and Technology, Wenzhou-Kean University, Wenzhou, China; ^4^ Wenzhou Municipal Key Laboratory for Applied Biomedical and Biopharmaceutical Informatics, Wenzhou-Kean University, Wenzhou, China; ^5^ Zhejiang Bioinformatics International Science and Technology Cooperation Center, Wenzhou-Kean University, Wenzhou, China; ^6^ Shenzhen Huayuan Biological Science Research Institute, Shenzhen Huayuan Biotechnology Co. Ltd., Shenzhen, China; ^7^ Center for Molecular Medicine and Genetics, School of Medicine, Wayne State University, Detroit, MI, United States

**Keywords:** breast neoplasms, long non-coding RNAs, non-coding RNAs, precision medicine, radioresistance, radiosensitivity, radiotherapy, genomics

## Abstract

Radiotherapy (RT) serves as one of the key adjuvant treatments in management of breast cancer. Nevertheless, RT has two major problems: side effects and radioresistance. Given that patients respond differently to RT, it is imperative to understand the molecular mechanisms underlying these differences. Two-thirds of human genes do not encode proteins, as we have realized from genome-scale studies conducted after the advent of the genomic era; nevertheless, molecular understanding of breast cancer to date has been attained almost entirely based on protein-coding genes and their pathways. Long non-coding RNAs (lncRNAs) are a poorly understood but abundant class of human genes that yield functional non-protein-coding RNA transcripts. Here, we canvass the field to seek evidence for the hypothesis that lncRNAs contribute to radioresistance in breast cancer. RT-responsive lncRNAs ranging from “classical” lncRNAs discovered at the dawn of the post-genomic era (such as HOTAIR, NEAT1, and CCAT), to long intergenic lncRNAs such as LINC00511 and LINC02582, antisense lncRNAs such as AFAP-AS1 and FGD5-AS1, and pseudogene transcripts such as DUXAP8 were found during our screen of the literature. Radiation-related pathways modulated by these lncRNAs include DNA damage repair, cell cycle, cancer stem cells phenotype and apoptosis. Thus, providing a clear picture of these lncRNAs’ underlying RT-relevant molecular mechanisms should help improve overall survival and optimize the best radiation dose for each individual patient. Moreover, in healthy humans, lncRNAs show greater natural expression variation than protein-coding genes, even across individuals, alluding to their exceptional potential for targeting in truly personalized, precision medicine.

## 1 The intersection of radiotherapy and long non-coding RNAs in breast cancer

Radiotherapy (RT) serves as one of the adjuvant treatment modalities in the control of many malignancies including breast cancer. The other modalities are chemotherapy, hormone therapy, immunotherapy, and targeted therapy (1–3). Along with surgery and chemotherapy, RT significantly reduces the risk of recurrence and improves overall survival in breast cancer patients (4, 5). 

However, breast cancer is heterogeneous in terms of its genetics and clinical characteristics, and this heterogeneity is classified through distinct subtypes. While 5-10% of breast cancers are due to inherited disease causative alleles and mutations in the BRCA1 and BRCA2 genes (6, 7), the vast majority of cases have a more complex etiology that is not confined to genetics and does not involve major single-gene risk factors. Breast cancer is traditionally classified into four groups based on immunohistochemical expression of hormone receptors: estrogen receptor positive (ER+), progesterone receptor positive (PR+), human epidermal growth factor receptor positive (HER2+), and triple-negative (TNBC), characterized by the absence of any of the above receptors. However, in light of a deeper understanding of breast cancer biology at the molecular level and by considering gene expression profiles, breast cancer can be stratified into four primary groups as luminal A, luminal B, HER-2, basal, and also a normal-breast-like group which closely resembles luminal A subtype (8, 9). While research over the past decade has focused primarily on protein-coding genes, the importance of non-coding regions of the genome cannot be underestimated.

Beyond protein-coding genes, non-coding RNAs (ncRNAs) constitute a significant portion of the human transcriptome (99% of all RNAs in a cell (10), and encoded by >65% of all genes), providing a promising horizon to capture key regulators in the cancer gene networks. It is well documented in the literature that long non-coding RNAs (lncRNAs), as a subset of ncRNAs, play a major role in the pathogenesis of breast cancer (11). In our previous work, we demonstrated that estrogen-induced/repressed lncRNAs serve as key oncogenes or tumor suppressors in breast cancer (12). Nevertheless, little remains known about how lncRNAs are functionally pertinent to radiation response, motivating this review. Beyond breast cancer, mounting evidence points to a direct functional association between Y chromosome-encoded lncRNAs and radiation sensitivity in male non-small cell lung cancer (NSCLC) (13). In our comprehensive exploration, we seize the opportunity to provide a briefing about radiotherapy in breast cancer management to accommodate readers unacquainted with this specialized field. Additionally, we recap how the cell reacts to the radiation at the molecular level and what the consequences of the exposure are: apoptosis, necrosis, necroptosis, pyroptosis, ferroptosis, cuproptosis, autophagy, senescence, and mitotic catastrophe (14). MicroRNAs in breast cancer are already well-characterized; this review hence will not pursue that topic. Instead, we delve into the world of lncRNAs due to their important role in the genome, offering a concise introduction for those seeking general information. Next, we tabulated the results of PubMed search for full-text articles with the following search terms:{“radioresistant” and/or “radiosensitive”} and “breast cancer” and (“lncrna” and/or “lincrna”). Finally, we reiterate how parallel development of chemical modifiers and technological breakthroughs across the wide spectrum of clinical approaches, from imaging technology to high-throughput omics, and with special relevance to the role of lncRNAs in radiosensitivity and resistance, impacts the field of RT in a way that will empower personalized medicine.

### 1.1 Understanding radiotherapy in breast cancer: protocols, challenges, and molecular insights

#### 1.1.1 Current standards for breast cancer radiotherapy

Various radiotherapy protocols are available for RT of breast cancer patients. The standard of care is to deliver a total radiation dose of 50 Gray in 25 fractions of 2.0 Gray over 5 weeks. Nevertheless, the current guidelines of the American Society for Radiation Oncology (ASTRO) recommend a hypofractionated (or accelerated) regimen for whole-breast radiation therapy in which patients receive the same total dose fractionated over a shorter period of time (15). A radiation oncologist selects the appropriate fractionation schedules according to the clinical features and histopathologic characteristics of patients (16). **Table 1** summarizes the guiding principles of RT for breast cancer.

**Table 1:** Radiotherapy for breast cancer.

Radiation therapy aspects	Details and effects
**Types of radiation beam**	*Photon (e.g., X-rays) and proton
**Delivery methods**	**External radiation (Teletherapy)
	Internal radiation (brachytherapy)
**Who receives radiation in breast cancer**	***Radiation after lumpectomy
	****Radiation after mastectomy
	Radiation for locally advanced breast cancer
	Radiation for managing metastatic breast cancer
**Side effects from radiation therapy**	Common: Mild to moderate fatigue, skin irritation such as itchiness, redness, peeling or blistering, breast swelling, changes in skin sensation. Depending on which tissues are exposed, radiation therapy may cause or increase the risk of arm swelling (lymphedema) if the lymph nodes under the arm are treated; damage or complications leading to removal of an implant in women who have a mastectomy and undergo breast reconstruction with an implant; rib fracture; or chest wall tenderness. Rare to very rare: Inflamed lung tissue or heart damage, secondary cancers, such as bone or muscle cancers (sarcomas) or lung cancer.
**Types and schedules**	For external beam: Whole breast radiation Accelerated partial breast irradiation Chest wall radiation Lymph node radiation For Brachytherapy: Intracavitary brachytherapy Interstitial brachytherapy

*X-rays, a highly penetrating electromagnetic radiation, is commonly applied. The radiation oncologist generally uses a medical linear accelerator (LINAC). It is a machine that delivers high-energy X-rays or electrons to the malignant tumor. Modern LINACs ensure a more precise delivery of RT to tumors while minimizing the dose to surrounding normal tissue. Cyclotron and synchrotron are the most common machines that generate and accelerate protons. Proton therapy is called heavy ion therapy as well. The protons damage the cells in the same way as photon therapy but with less damage to vital organs as the lung and heart. However, proton therapy is expensive and currently applied in clinical trials.

**There are two modes to deliver radiation into the tumor site. For external radiation therapy, radiation is projected by a machine located outside the body. For the internal mode of delivery, equipment such as seeds, ribbons, and capsules that contains a radiation source is implanted inside the body adjacent to the tumor site.

***Lumpectomy or breast-conserving surgery is a treatment option for early-stage breast cancer. Only abnormal tissues along with the small surrounding normal tissues are excised.

****In mastectomy, the whole breast is removed.

The content of this table is adapted from: https://www.breastcancer.org/treatment/radiation-therapy/side-effects.

#### 1.1.2 Challenges in breast cancer radiotherapy: side effects and radioresistance

Though highly effective, RT as an approach to treating cancer has two problems: side effects and radioresistance. The most common short-term side effects are fatigue, swelling in the breast, skin changes in the treated area similar to a sunburn (redness, skin peeling, darkening of the skin), or other serious long-term complications as lymphedema, shoulder stiffness, brachial plexopathy, predisposition to rib fracture and angiosarcoma, a rare type of cancer (17). Locoregional recurrence following RT could be attributed to intrinsic radioresistance or the development of *de novo* resistance features in a particular subpopulation (18). Moreover, different molecular subtypes of breast cancer do not respond equally well to RT, in which luminal cancers, in particular, luminal subtype A, benefit the most compared to HER2-positive and TNBC (19).

#### 1.1.3 Cellular and molecular responses to radiotherapy in breast cancer

In the context of RT, cell fates and molecular events play a crucial role in the response to treatment. RT harnesses the intense energy of photons to target cancerous cells that persist after surgery, potentially aiding in the cure of metastases. Mechanisms of action include but are not limited to: direct, by induction of DNA damage and subsequent cell death; or indirect, by targeting tumor microenvironment through vascular damage or modulation of anti-tumor immune responses (20–22).

Following delivery at the tumor site, high-energy ionizing radiation directly breaks DNA, and also decomposes water into free radicals (water radiolysis) thus indirectly damaging DNA. Various DNA lesions occur as a result, including: base damage, single-strand breaks (SSBs), double-strand breaks (DSBs), and intra as well as inter DNA crosslinks. As an aftermath of severe damage, cell death occurs. Mitotic catastrophe and mitotic death, apoptosis, necrosis, senescence, autophagy, and necroptosis and ferroptosis are among the currently recognized types of radiation-induced cell death (4). Nevertheless, cancerous cells are not the only cells that are affected by RT, since RT does not distinguish between cell types. These side effects of RT occur systematically and locally on the normal cells nearby the tumor site that is being treated. Due to the proximity of the lung, heart, and contralateral healthy breast to cancerous breast tissue, and also blood exposure to RT, complications can arise (23). Moreover, each tumor resides in a field of non-cancerous cells, such as stromal cells which could govern radiotherapy responsiveness. The dynamic crosstalk of these neighboring cells with tumors within the tumor microenvironment makes them a potential modulator on radiation response. The key types of stromal cells include immune cells, cancer-associated fibroblasts (CAF), extracellular matrix, endothelial cells, and adipocytes. They promote radioresistance by adopting cancer stem cell properties, providing the secreted pro-survival factors, affecting metabolites and oxygen availability, angiogenesis, and immunomodulatory effects. Hence, besides the tumor-intrinsic factors, the tumor microenvironment and host immune system are notable factors affecting RT response (24).

Upon irradiation-induced damage, DNA damage responses (DDRs) are invoked. The key proteins Ataxia-Telangiectasia Mutated (ATM) and Ataxia-Telangiectasia and Rad3-Related protein (ATR) sense the damage and trigger the DNA damage response. Depending on the cell cycle stage, DNA double-strand lesions are repaired by non-homologous end joining (NHEJ) and homologous recombination (HR). The same protective DDRs safeguard tumor cells against radiation-induced cell death. Thus, radiation-induced DNA lesions and protective DDRs are, essentially, double-edged swords: they act in a way that develops, in parallel, dysfunctional normal tissue and cell damages as well as radioresistant features. Hence, a major consideration in RT is how to tip this balance in favor of the patient, by therapeutic approaches that minimize toxicity in normal tissues (e.g., by the development of mechanistically-driven radioprotectors, or techniques to more precisely deliver radiation to tumor site) and to better sensitize tumor cells to radiation (e.g., by the development of radiosensitizers, or targeting DDR signaling pathways to heighten tumor radiosensitization).

While radiation can damage DNA irreparably by affecting cell organelles, cell membrane properties, signal transduction, tumor cell phenotype, and the tumor immune response (25), cancerous cells can still evade lethal DNA damage by activating efficient DNA repair mechanisms. A growing list of lncRNAs facilitates various steps of the DNA repair from the detection of DNA lesions to activating signaling pathways that initiate DNA repair processes (26). The lncRNA GUARDIN is noteworthy for its association with BRCA1 and BARD1, which participate in a variety of DNA damage response pathways (DDRs) (27). This is merely the proverbial “tip of the iceberg.” There are definitely many other lncRNAs interacting with DNA damage repair and hence radiation response genes. Identification of any radiation-induced or suppressed lncRNAs would have practical implications for sensitizing cancer cells to RT. Emerging evidence unequivocally indicates that there is a huge reservoir of yet-unknown oncogenes and tumor suppressors in lncRNA data, including specifically in breast cancer (12), and undoubtedly many of these will be relevant to radiation response.

Considering the marked differences between patients in response to RT, and the associated toxicity and side effects, it is of utmost importance to decipher the complex molecular networks that are responsible for these differences and for the mechanisms leading to radioresistance and treatment failure. Providing a clear picture of the underlying molecular mechanisms could help improve overall survival and to optimize the best radiation dose for any individual, in a clear application of personalized medicine. Accordingly, the present review aims to summarize all the efforts of the last two decades (~2000-2022) to unravel radiation-induced pathways with a focus on breast cancer, specifically on the role of lncRNAs therein.

### 1.2 LncRNAs: emerging players in genome regulation

#### 1.2.1 Unraveling the genomic landscape: LncRNAs in focus

Completion of the Human Genome Project (HGP) in 2003 spurred the emergence of disciplines and projects aimed at elucidating genome functionality. The Encyclopedia of DNA Elements (ENCODE) and the Functional Annotation of the Mammalian Genome (FANTOM) consortia, as HGP successors, demonstrated that 80% of the genome is functional (28) and that mammals have more non-coding RNA genes than protein-coding genes (29), respectively. The number contrasts with the mere 1.5% of the genome that is occupied by exons of protein-coding genes. Formerly considered to be “junk DNA,” the non-coding part of the genome is now widely understood to contain regulatory elements and non-coding RNA genes. Much of this non-coding DNA is transcribed into non-coding RNAs (ncRNAs), including micro and macro noncoding RNAs. Among these ncRNAs, lncRNAs represent the most prevalent and functionally diverse class. They have been increasingly highlighted as transcripts with emerging roles in crucial aspects of biological processes. It is estimated that they are pervasively transcribed from 15,000 to 80,000 distinct loci in the human genome (30–32). LncRNAs are defined as transcripts of more than 500 nucleotides in length, lacking open reading frames, and transcribed by RNA Polymerase II. They can interact with genomic DNA, RNA partners, as well as with RNA binding proteins. LncRNAs are involved in target-specific as well as in global regulatory mechanisms, and are ubiquitously transcribed (10).

#### 1.2.2 LncRNAs: architects of evolution and cellular identity

The numbers of lncRNAs, unlike protein-coding genes, correlate with species complexity during evolution, and approximately 60-75% of human lncRNAs are primate-specific (32–34). They may have appeared and increased in their numbers in ancestral mammalian species soon after the mammalian radiation, conferring higher levels of complexity and gene regulation (32, 35) that may have even led to the development of evolutionarily new organs (for example, chimpanzees have entire organs – such as the vomeronasal organ – that are absent in humans, whereas human and gorilla RNAs in the brain show highly discrepant splicing (36)). While the knowledge of primary amino acid sequence and homology modeling greatly enhanced the ability to predict two or three-dimensional structures of proteins and hence their function, this has not been the case for lncRNAs since most RNA structural biology has historically focused on ribosomal RNAs and other “classical” short RNAs (37). Despite the challenges associated with *in silico* prediction methods for deciphering structural/primary sequence correlations, the concept of a “modular RNA regulatory code” arose due to evidence that secondary structures in lncRNAs remain conserved in orthologs between mammalian species despite an absence of sequence conservation (38). The latter makes lncRNAs “invisible” to sequence homology-based bioinformatic tools used for protein-coding genes. It is therefore possible to predict lncRNAs’ modes of action by mapping higher-order structural features and functional networks rather than their primary sequences. Genetic experiments could help determine whether their regulatory function is modularity-dependent and/or independent (38). Furthermore, we expect artificial intelligence (AI) to make a measurable impact both in the area of predicting lncRNA secondary structure and in detecting cryptic cross-species lncRNA homologies.

As a result of the evolutionary plasticity of these sequences, it is increasingly posited that the genome was liberated from the rigidity of mechanisms driven by highly conserved coding genes, allowing for new functions to be developed. In contrast to mRNAs, lncRNAs usually exhibit restricted and tissue-specific expression patterns (32, 39) and are often cell-type-specific, which suggests they are involved in cell state and developmental pathway regulation (40). In addition, most are located in the nucleus, but a significant proportion is in the cytoplasm (10). The cytoplasmic proportion is estimated to be 75% in human and *Drosophila* cells (41). Consistent with these results, in *Drosophila* models, RNA FISH revealed that more lncRNAs were found in the cytoplasm (40%) than in the nucleus (4%) (42).

Similarly to mRNAs, most cytoplasmic lncRNAs are spliced, polyadenylated, and 7-methylguanosine-capped. Based on the genomic positions of lncRNA-encoding loci relative to nearby or overlapping protein-coding genes, lncRNA genes can be classified as intergenic, antisense, intronic, and/or overlapping in other ways, relative to those coding genes (10).

#### 1.2.3 Functional characteristics of LncRNAs

**Figure 1:**
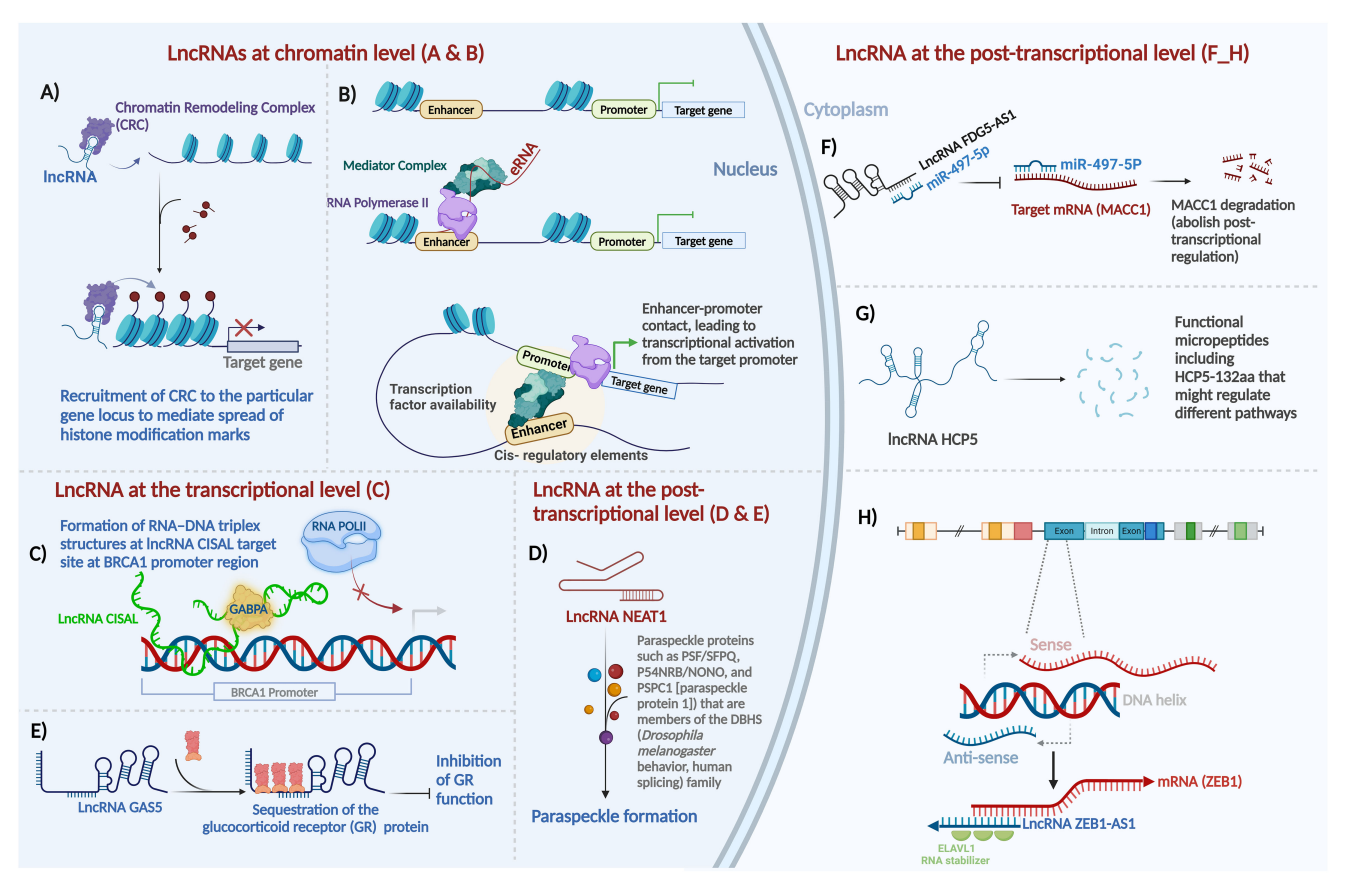
Major mechanisms of action of long non-coding RNAs in mammalian systems. Gene expression is regulated at three distinct levels - epigenetic (chromatin), transcriptional, and post-transcriptional - by lncRNAs as shown here and as detailed in the main text. **(A)** guide lncRNA, **(B)** enhancer RNA, **(C)** forming a triplex structure with DNA sequence at regulatory regions and interaction with transcription factors to regulate gene expression, **(D)** lncRNA as scaffold, **(E)** lncRNA as decoy, **(F)** lncRNA as sponge for miRNAs, **(G)** lncRNA-encoded protein, and **(H)** mRNA:lncRNA hybridization in post-transcriptional sense-antisense pairs. Most of these effects can be classified as epigenetic and transcriptional regulation, as illustrated in the nucleus on the left, or post-transcriptional mechanisms in the cytoplasm, on the right. For details about lncRNA function at the chromatin level, the transcriptional level, and the post-transcriptional level refer to the main text. Created with BioRender.com.

LncRNAs regulate gene expression at multiple levels, including chromatin organization, transcriptional and post-transcriptional regulation (**Figure 1**).

**LncRNAs at the chromatin level:** LncRNAs can modulate gene expression through deposition of epigenetic modifiers at particular regions of the genome by acting either in cis or in trans. For the purposes of this review, we define “cis” regulation as that occurring within the same locus, for example when an antisense lncRNA regulates its counterpart sense mRNA transcribed from the opposite direction in the same locus (regardless of allele specificity), and we define “trans” regulation as any scenario where the lncRNA and its target are encoded at different loci (43). Exemplifying this regulatory modality, the lncRNA HOX antisense intergenic RNA (HOTAIR), among the first oncogenic lncRNAs identified in primary and metastatic breast tumors (44), serves as a platform for the assembly of two distinct histone modifiers. Through the recruitment of Polycomb Repressive Complex 2 (PRC2) and LSD1–CoREST, lncRNA HOTAIR facilitates H3K27 trimethylation (45) and H3K4 demethylation (46), respectively, to repress gene expression (**Figure 1A**). In a contrarian fashion, the upstream master lncRNA of the inflammatory chemokine locus (UMLILO) activates expression of its target gene through recruitment of activating factors, such as the WD repeat-containing protein 5 (WDR5)–mixed lineage leukemia protein 1 (MLL1) (WDR5–MLL) complex, to the target promoter enabling their H3K4Me3 epigenetic priming (47). In addition to recruiting epigenetic modifiers, lncRNAs can induce chromatin loop formation to reformat the genome. For instance, ncRNA-a7, member of a class of lncRNAs involved in long-range transcriptional activation through the association with the Mediator complex, forms a co-activator complex to form a loop with its target locus (**Figure 1B**) (48).

**LncRNAs at the transcriptional level**: LncRNAs also function positively and negatively - depending on the specific lncRNA, the target/s, and the cellular and network contexts - as transcriptional regulators in many cases. LncRNAs can directly interact with transcription factors along the genomic DNA to induce or suppress transcription. The mechanisms of actions include but are not limited to several key modalities. By preventing transcription initiation complex formation at promoters, lncRNA may suppress transcription through RNA–DNA triplex formation. The regulatory outcome in this case is that lncRNAs sequester transcription factors from their cognate DNA-binding sites. For instance, through direct binding to the BRCA1 (breast cancer early onset 1, the first breast cancer gene that was ever discovered) promoter and forming a tertiary structure, cisplatin-sensitivity-associated lncRNA (CISAL) sequesters the BRCA1-activating transcription factor GABPA away from downstream regulatory regions (**Figure 1C**) (49). LncRNAs also activate transcription by recruiting transcription factors to the targeted promoters or acting as transcription factor co-activators. LncRNA DLEU1 (deleted in lymphocytic leukemia 1) acts as the coactivator for hypoxia inducible factor 1 subunit alpha (HIF-1α) to induce expression of cytoskeleton associated protein 2 (CKAP2) and consequently pro-tumor activities in breast cancer (50). Additionally, a number of lncRNAs regulate transcription by controlling nucleocytoplasmic transport of transcription factors. For instance, the lncRNA non-coding repressor of NFAT (NRON) inhibits nuclear translocation of dephosphorylated nuclear factor of activated T cells (NFAT) trans-activator by interacting with importin-beta family members (51).

**LncRNAs as regulators at the post-transcriptional level:** Post-transcriptionally, lncRNAs exhibit distinct mechanisms, which are diverse and protean in their versatility - in contrast to the mono-mechanistic nature of microRNAs, which act almost solely as post-transcriptional suppressors.

LncRNAs serve as architectural scaffolds for assembling proteins to enable biological events, such as the formation of key nuclear subcompartments, in particular paraspeckles, containing the lncRNA NEAT1 (**Figure 1D**) (52), as well as determinants of chromosome structure in the interphase nucleus, including long-range interchromosomal interactions (53), regulators of telomere activity such as TERRA (telomeric repeat-containing RNA) (54); and essential accessories of the actin cytoskeleton (55).

LncRNA-protein interactions comprise an additional important aspect of lncRNA-mediated gene regulation. LncRNAs act through direct functional interactions with specific proteins, forming lncRNA ribonucleoprotein complexes (lncRNPs), such as the complex that allows the BRCA1 protein in breast cancer to function upon activation by a direct-binder primate-specific lncRNA. For instance, as a p53-responsive lncRNA, GUARDIN acts as a binding platform joining the breast cancer early onset protein BRCA1 and its partner BARD1, which cooperate to stimulate cell proliferation and survival (27). Another example of an lncRNA that interacts with proteins to accomplish its function in breast cancer is the lncRNA LINP. It contributes to radioresistance in TNBC by stabilizing Ku80 and DNA-PKcs complexes after double-strand DNA breaks (DSB) (56).

One important subtype of lncRNAs that act through lncRNP formation is lncRNA which directly binds transcription factor proteins. In this context, lncRNAs directly interact with transcription factors and serve as their co-activators or co-repressors. For instance, the glucocorticoid response element (GRE)-like element ribomimic sequence, found within the lncRNA growth arrest-specific 5 (GAS5), mimics the consensus genomic-DNA binding site motif that GR recognizes, and as a result, the element serves as a decoy to repress glucocorticoid receptor (GR) by titrating bioavailable GR molecules out of the pool that is available to bind to GR-responsive promoters (**Figure 1E**) (57). Through such protein binding that leads to inactivation or sequestration, lncRNAs inhibit the function of proteins.

MicroRNAs can bind lncRNAs based on Watson-Crick sequence complementarity. As a result of sponging microRNAs, lncRNAs may rescue the half-life, stability, or translation of the mRNAs cognate to the affected microRNAs. LncRNAs act as competing endogenous RNAs (ceRNAs) if they bind miRNAs that are hence prevented from binding the mRNAs that they would otherwise be suppressing. For instance, the lncRNA FYVE RhoGEF and PH Domain containing 5 antisense RNA 1 (FGD5-AS1) promotes the radioresistance of breast cancer cells in an FGD5-independent manner, by sponging miR-497-5p, which in turn results in the upregulation of mir-497-5p’s target mRNA, metastasis-associated in colon cancer 1 (MACC1) (**Figure 1F**) (58). MACC1 was first discovered in colon cancer (59). As an oncogene, it plays an important role in tumor invasion and metastasis in a wide range of solid tumors, primarily by regulating genes that contribute to the epithelial-mesenchymal transition (EMT) (60).

In addition to their multiple functional modalities that we have thus far discussed, lncRNAs often contain cryptic short open reading frames (ORFs) that can encode micropeptides, allowing the lncRNAs to be bifunctional through distinct RNA-based and peptide-based roles. Despite early assumptions that most lncRNAs are genuinely noncoding and lack even short ORFs that could be translated by ribosomes to yield peptides, protein-coding capabilities of a small but reproducible subset of lncRNAs were discovered by us (61) and subsequently confirmed by numerous groups. For example, in breast cancer, the LINC00908-encoded polypeptide ASRPS (a small regulatory peptide of STAT3) (62), the lncRNA MAGI2-AS3-encoded polypeptide (63), and the lncRNA HCP5-encoded peptide (64) have been proven to have functional roles in breast cancer pathogenesis (**Figure 1G**).

Direct relationships of lncRNAs to mRNAs in ceRNA networks are well-documented (65). Antisense lncRNAs generally regulate sense protein-coding transcripts in two ways: cis and trans (29). A major and frequent mechanism of lncRNA post-transcriptional action is antisense regulation of cognate protein-coding mRNAs encoded on the opposite strand of the same locus; this can be positive or negative, depending on the specific sense-antisense pair (29, 66). Sense/antisense, coding/noncoding, mRNA/lncRNA pairs are a prevalent phenomenon in the human transcriptome with demonstrated functional importance in breast cancer. For instance, lncRNA ZEB1-AS1 positively regulates ZEB1 expression. By binding to embryonic lethal vision-like protein 1 (ELAVL1), ZEB1-AS1 stabilizes ZEB1 mRNA, facilitating the progression of TNBC (**Figure 1H**) (67). Breast cancer literature already demonstrates the prevalence of sense/antisense gene pair regulation by lncRNAs in mRNA/lncRNA pairs, such as PDCD4/lncRNA PDCD4-AS1 (68), ZNRD1/lncRNA ZNRD1-AS1 (69), HMMR/lncRNA HMMR-AS1 (70), HYOU1/lncRNA HYOU1-AS (71), and HIF-1α/lncRNA HIF-1α-AS (72).

Beside overlapping-gene regulation by antisense lncRNAs, they can regulate genes in *trans* and at distant loci. Certain lncRNAs partially pair with target mRNA 3’UTRs through their Alu elements and activate STAU-mediated mRNA decay (73); these Alu-Alu sense-antisense mechanisms are repeat-mediated and, unlike more common antisense regulatory modalities, generally involve transcripts from different loci, rather than from opposite strands of the same locus.

Keeping with the broad theme of sequence-mediated regulation of downstream effectors of lncRNAs, pseudogene transcripts are another abundant class of lncRNAs and have been implicated in the regulation of mRNA transcripts of the pseudogenes’ parental genes, through a protean variety of versatile mechanisms which include but are not limited to epigenetic feedback to the parental gene promoter as well as ceRNA networks dependent on the pseudogenes’ sponging of the miRNA regulators of the parental genes. Pseudogene-derived functional lncRNAs are now well-documented, including as fundamental regulators of the expression of the cognate protein-coding genes (74, 75). Recent studies suggest that pseudogenes regulate gene expression, in part, by being processed into short interfering RNAs that regulate coding genes, as well as, in other cases, by acting as microRNA decoys to regulate tumor suppressors and oncogenes (76). The lncRNA DUXAP8, transcribed from a pseudogene, modulates both the P3K/AKT/mTOR pathway and the EZH2-E-cadherin/RHOB pathways to exert its role in inducing breast cancer radioresistance (77).

## 2 Long non-coding RNAs of known functional relevance to radiation response in breast cancer

The role of lncRNAs in cancer drug resistance is now well-established (78). The emerging evidence suggests the involvement of lncRNA in RT response. Dissecting the underlying mechanisms in radioresponsiveness can provide predictive biomarkers of radioresponsiveness and identify functional molecules in radiation response pathways that will contribute to the development of targeted radiotherapies (79). **Table 2** summarizes the key literature of the past 20 years on lncRNAs involved in radiation response in breast cancer.

**Table 2:** LncRNAs involved in RT response in breast cancer.

NO	LncRNA	1-Cell lines 2-Animal model 3*-In vivo* and *in vitro* tissues/specimens	Main finding	The associated pathway(s)/axis/The biological effect that was assessed	REF
**1**	AFAP1-AS1	• MDA-MB-231 • Balb/c mice • TNBC/tissue samples (N=125)	lncAFAP1-AS1 serves as a key player in inducing radioresistance of TNBC via activating the Wnt/b-catenin signaling pathway.	Wnt/b-catenin signaling pathway	(80)
**2**	CCAT1	• MCF-7 and MDA-MB-231 • BC radio resistance and Sensitive/N=65	CCAT1 knockdown could dampen radioresistance by regulating miR-148b	CCAT1/miR-148b proliferation and apoptosis	(81)
**3**	DUXAP8	• MCF-12A, MCF-12 F, MCF-7, T47D, ZR-75-1, HCC-1806, MDA-MB-468, BT-549, and MDA-MB-231, and the normal mammary epithelial cell line MCF-10A • BC and normal adjacent (N=50)	DUXAP8 activates P3K/AKT/mTOR pathway and suppress EZH2-E-cadherin/RHOB axis, to promote breast cancer cell resistance to radiation	PI3K/AKT/ mTOR and EZH2-E-cadherin/RHOB pathways	(77)
**4**	FGD5-AS1	• MCF-7 and MDA-MB-231 • BC and paired normal tissues (N=50)	Mechanistically, it was shown that FGD5-AS1 depletion dampen/attenuate radioresistance through the inhibition of MACC1 by competitive sponging of miR-497-5p	FGD5-AS1/miR-497-5p/MACC1 Apoptosis	(58)
**5**	LINP1	• MDA-MB-231, MDA-MB-468, MCF7	LINP1 promotes NHEJ-mediated DNA repair after double-strand DNA break (DSB) by stabilizing Ku80 and DNA-PKcs. An RNA-based therapeutics based on LINP1 knockdown could sensitize tumors to irradiation	p53/mir29/LINP1 and EGFR/RAS-MEK-ERK/LINP1 NHEJ pathway	(56)
**6**	LINC00511	• MDA-MB-231, • MDA-MB-436, MDA-MB-361, MCF-7 and breast • epithelial cell MCF-10A • 5-week-old nude mice	LINC00511/STXBP4/mir-185 axis modulates radioresistance in breast cancer LINC00511 up-regulates STXBP4 expression by competitive sponging of miR-185 resulting in radioresistance	LINC00511/STXBP4/mir-185 Proliferation and apoptosis	(82)
**7**	LINC02582	• MCF-10A, MDA-MB-231, MCF-7, BT549, SKBR3, T47D, and BT474 • 136 FFPE and 44 fresh frozen samples	Through interaction with USP7, LINC02582 deubiquitinates and stabilizes checkpoint kinase 1 (CHK1), stimulating radioresistance. LINC02582 is a direct downstream target of miR-200c	miR-200c/LINC02582/USP7/CHK1 DNA damage response	(83)
**8**	NEAT1	• MDA-MB-231 and Hs578t	NEAT1 maintains the CSC population, hence enhances the radioresistance attributed to cancer stem cells. A potential radiosensitizing effect can be achieved by the administration of exogenous NQO1 substrates.	Cancer stem cells activity and cell growth of whole populations of cancer cells	(84)
**9**	HOTAIR	• T47D, MCF-7, SKBR3, BT549, MDAMB231and MCF-10A	HOTAIR promotes radioresistance in MDA-MB231 breast cancer cells and accelerates proliferation through the Akt pathway by targeting HOXD10	PI3K/AKT-BAD	(85)
		• MCF-7, SKBR3 and MDA-231 • Paired breast cancer tissues and adjacent normal tissues (N=10)	By inhibiting HOTAIR lncRNA, miR-218 is released and radiosensitivity is induced.	HOTAIR-miR-218 DNA damage and cell cycle arrest/apoptosis	(86)
		• MCF-7, T47D, LM-MCF-7, BT-474, SKBR-3 and MDA-MB-231 • breast cancer and normal adjacent (N=20)	HOTAIR sponges miR-449b-5p resulting in increased expression of HSPA1A, conferring radioresistance in breast cancer	HOTAIR/miR-449b-5p/HSPA1A Cell proliferation and tumor growth	(87)
		• MCF-7/MCF10A • BALB/c nude mice • Invasive ductal carcinoma (IDC) and normal adjacent (N=40)	The overexpression of HOTAIR stimulates the expression of DNA damage repair factors including KU70 and KU80, DNA-PKs, and ATM	HOTAIR/KU70, KU80, DNA PKs, ATM Cell cycle, Apoptosis, Cell proliferation, Tumor size	(88)

Among these are classical (discovered during the early era of the field and now well-understood) lncRNAs such as CCAT1 (81), NEAT1 (84), and HOTAIR (85–88). The aberrant expression of HOTAIR (89), NEAT1 (90), and CCAT1 (91) is implicated in breast cancer pathogenesis. Additionally, a number of gene-desert long intergenic non-coding RNAs (lincRNAs) including LINC00511 (82) and LINC02582 (83) have been discovered during this screen. Furthermore, there are several lncRNAs highlighted in the literature relevant to RT whose genomic position provides immediate clues to their function and thereby facilitates specific therapeutic targeting or rescue approaches, including AFAP-AS1 (80) and FGD5-AS1 (58). A number of cancers are pathoetiologically linked to the lncRNA actin filament-associated protein 1 antisense RNA1 (lncAFAP1-AS1). Its second exon overlaps with exons 14-16 of the AFAP1 gene on 4p16.1. Via AFAP1-dependent and independent activities, it affects the signaling pathways involved in migratory potential and metastatic activities including PI3K/AKT, Wnt/b-catenin, EGFR/AKT, PTEN/pAKT, and RhoA/Rac2. In general, AFAP1-AS1 is considered an oncogenic lncRNA. AFAP1-independent mechanisms mediate most of the effects exerted by this lncRNA during carcinogenesis (92, 93). It has also been shown that AFAP1-AS1 induces EMT through the influence of Wnt/b-catenin signaling, not only in TNBC cells (94), but also in tongue squamous cell carcinoma (95), osteosarcoma (96), colon cancer (97), and cervical cancer (98). There is now evidence that AFAP1-AS1 is involved in promoting TNBC radioresistance via activation of the Wnt/b-catenin pathway (80).

The FGD5-AS1 lncRNA is antisense to the FGD5 gene whose direct role in breast cancer (99) gives us an immediate clue concerning the potential use of this RNA as a target for frontline therapy. We showed nearly two decades ago that antisense lncRNAs are key regulators in cancer models (29). Various cancers have abnormally high FGD5-AS1 expression that correlates with lymph node metastasis, tumor invasion, survival time, and recurrence rate. FGD5-AS1 stimulates cancer cell proliferation, metastasis, invasion, and chemoresistance both *in vitro* and *in vivo* by competing with microRNAs (including miR-5590-3p, miR-129-5p, miR-196a-5p, and miR-142-5p), leading to the mRNA’s stability and hence cell growth (100). Its relevance to radiation response through the FGD5-AS1/miR-497-5p/MACC1 axis (58) suggests that it is potentially a target in radiation sensitization. Therefore, FGD5-AS1 is an extraordinary sponge and sink for at least five different microRNAs that, in its absence, may downregulate the FGD5 mRNA. Furthermore, here for the first time we canvassed the literature to show that the expressed pseudogenes such as DUXAP8 are relevant not just to breast cancer (101) but to radiotherapy against breast cancer (77).

Therefore, we presented evidence that these dispersedly studied RT-associated lncRNAs are involved in the same biological processes including but not limited to DNA damage repair, cell cycle, cancer stem cells phenotype, and apoptosis.

## 3 Development of non-invasive radio-responsiveness biomarkers from the circulating lncRNAome

The primary source that drives a molecular signature with the potential to predict or assess the prognosis of radio-responsiveness and adjust to the optimum radiation intensity for each patient is the breast tumor itself. Nonetheless, liquid biopsies (blood, urine, saliva, etc.) have long proven to be a useful surrogate reservoir for biomarker investigation. Blood, by having the advantage of being collected noninvasively, mirrors valuable information to assess a condition such as radiation response for a period of time, provided that the markers arising out of the tumor are expressed and correlated with the tumor properties. This helps continuous monitoring of patients over course of (pre-, on- or post-) treatment with the promising application for a metastatic disease where the metastatic site might not be detectable or accessible for examination.

Whole blood, and specific fractions such as peripheral blood mononuclear cells (PBMC), cell-free DNA, or exosomes, are all repositories of valuable biological data (102). Exosomes are nanosized vesicles, produced by all cell types and shed outside of the cells, that are an integral component of biofluids and hence are collected in any liquid biopsy. These vesicles contain DNA, RNA, protein, and metabolites which reflect the ongoing disease processes. These cargos correlate and change with disease conditions and hence are potential biomarkers that can track disease progression (103). Collectively, these information layers can be data-mined to define the best representative signature for accurate diagnosis, prognosis, and prediction, here in the context of radiation response (104). The ultimate goal of this effort is to eventually derive a “radiation fingerprint” capable of predicting a patient’s radiation response and adjusting the best radiation dose for any individual patient.

Currently, there are no clinically validated biomarkers to reliably guide optimal RT strategies. However, circular RNAs, including non-coding circRNAs, have emerged as stable and useful biomarkers in numerous types of cancer (105). Leveraging blood-derived biomarkers from non-invasive liquid biopsies allows real-time assessment of patient response. Given their advantages of early detection, noninvasiveness, and cost-effectiveness, developing circRNA biomarkers for radioresistance and radiosensitivity in breast cancer is a promising research avenue (106). Although efforts are underway, these biomarkers still require refinement and validation due to challenges related to sensitivity and specificity.

Breast cancer is complex and heterogeneous, with different pathway activations leading to varied oncogenic drivers even within the same subtype. These differences affect tumor responses to RT, and gene signatures may not fully capture this complexity. Validating gene signatures is challenging due to variations in treatment regimens, patient subtypes, and study designs. Considering the low expression level of lncRNAs in general, as well as differences in RNA extraction methods and gene expression analysis methods, also complicates the validation process. Translating lab research into clinical tests requires standard procedures, cost-effectiveness, and proof of improvement over existing practices. These challenges limit the clinical use of gene signatures in breast cancer treatment. Nonetheless, the scientific community remains optimistic about the impact of personalized medicine on breast cancer treatment in the coming decades (106).

**Figure 2:**
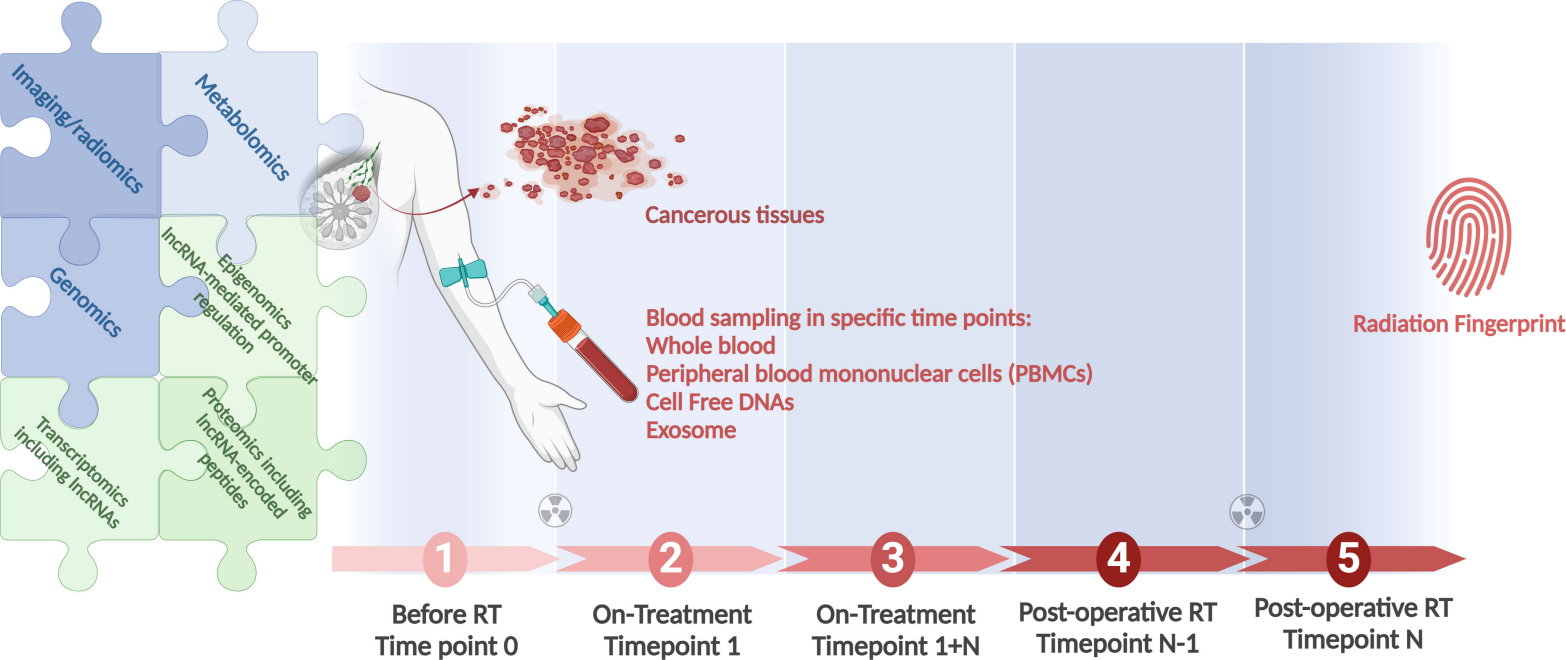
A workflow for integration of all clinical modules in RT, as genomics, lncRNA-inclusive transcriptomics, and radiomics, to identify the most informative radiation responsiveness signature. Longitudinal studies conducted in large cohorts provide the foundation. Associating patient genomics data with radiation response is at the focus of field radiation genomics. Areas dependent on lncRNAs, in view of these transcripts’ abundance, function, and prevalence, include epigenomics, transcriptomics, and proteomics. Correlating radiomics features with patient molecular profiles is the focus of the growing field of imaging genomics. Radiogenomics is a term that should be applicable for both the molecular and the imaging aspects of the field. Created with BioRender.com.

**Figure 2** depicts an integrated approach using the combination of all modalities to find the best informative model that could be applied individually to direct the RT.

## 4 Radiosensitizers and radioprotectors as potential regulators and targets of lncRNAs

Technological improvements in RT, including conformal radiotherapy (CRT), intensity-modulated radiotherapy (IMRT), image-guided radiotherapy (IGRT), and proton‐beam radiotherapy (PBT), have improved RT efficacy by precisely targeting the tumor and minimizing the collateral normal tissue damage. To make cancerous cells more vulnerable to RT and reduce the associated toxicity on normal cells, radiosensitizers and radioprotectors, respectively, have been developed (107).

Based on their structure, radiosensitizers are categorized into small molecules, macromolecules, and nanomaterials. A comprehensive list of radiosensitizers, and of the mechanisms through which radiosensitizers boost radiation response, has been developed (108). In general, they sensitize the tumors by enhancing ionizing radiation energy deposition, catalyzing reactive oxygen species (ROS) generation and subsequently reinforcing radiation’s damaging effect on biomolecules, and modulating the tumor microenvironment (TME) (108).

Radioprotectors, which are commonly antioxidants, minimize the deleterious effect of radiation when applied before or shortly after radiation therapy. To be adopted in clinical settings, radioprotectors should not possess protective effects on cancerous cells nor toxic effects on normal tissues and need to be conveniently administrated as well (109). Examples of radioprotectors include antioxidant compounds such as glutathione, lipoic acid, and the antioxidant vitamins A, C, and E, nitroxides, cysteine and cysteamine, melatonin, and novel radioprotectors as tetracyclines and fluoroquinolones. The most widely used US Food and Drug Administration (FDA) approved radioprotector is Amifostine (WR-2721), a systemically effective radiation countermeasure. However, the associated side effects have limited their application in all oncologic settings and highlight the unmet need for the development of novel protective compounds. A list of natural radioprotectors has recently been tabulated (110). A database of radiosensitizers and radioprotectors in which users can browse typical information of a desired compound has been reported as well (111). A curated database of validated radioprotectors is also available (112).

In view of the compelling evidence that lncRNAs contribute to radioresistance, as we have demonstrated here in the context of breast cancer, targeting them along with radiotherapy could potentially increase treatment effectiveness. An in-depth exploration of lncRNA-mediated regulation of radiosensitivity has highlighted potential radiation-related signaling pathways in various cancers (104, 113). In brief they exert their influence through intricate molecular mechanisms, including DNA damage repair, cell cycle regulation, apoptosis, modulation of cancer stem cells, EMT, and autophagy. However, additional basic and clinical studies are needed to understand the intricate interactions between lncRNAs and signaling molecules that affect radiosensitivity. Advancing the field necessitates the incorporation of both small and long RNAs in medium- to high-throughput screenings, aiming to replicate the effects of radioprotectors, thus underscoring the need for continued research in this area.

## 5 Personalized radiation oncology and how it relates to lncRNAomics

An ideal state of the field of radiation oncology could be envisaged when the right radiation dose could be applied for the right patient with maximum desired impacts and minimal side effects. This is a part of personalized medicine, where therapeutic plan, including radiation schedules, will be tailored with genetics and phenotypic features of each individual patient to achieve the most beneficial outcome. Up until now, the one-size-fits-all approach has been adopted and dose protocols have therefore been applied uniformly for all patients. Hence, RT-related side effects and resistance remain major challenges on the way of treating breast cancer. Due to the integral contribution of genetic background to individual radiation response as well as to the essential nature of medical imaging in clinical decision-making, here we discuss the complementary role of these frontiers in further detail.

Genetic background, along with clinical characteristics of patients such as histopathology and tumor grade, progression, and drug response profiles, holds useful clues for understanding the observed disparities in radioresponsiveness (114). The advent of high-throughput next-generation sequencing has paved the way to decipher this complexity at the genomic, transcriptomic, and epigenomic levels. In 2009, a Radiogenomics Consortium (RGC) was established to facilitate and promote multi-center collaboration of researchers linking genetic variants with response to radiation therapy.

A molecular radiosensitivity index (RSI) was developed to predict RT therapeutic benefit in two independent breast cancer datasets totaling 503 patients (115). The gene expression profile of 10 genes (*AR, cJun, STAT1, PKC, RelA, cABL, SUMO1, CDK1, HDAC1*, and *IRF1*) was assessed using a linear regression algorithm (115). Clonogenic survival assays coupled with gene expression subsequently facilitated the generation of a human breast cancer-specific radiosensitivity signature (Radiotype DX) with the potential to predict locoregional recurrence and personalized RT (116). Given that this signature is assessed in various solid tumors and is independent of molecular subtype in predicting local recurrence, it potentially could be adopted in various clinical contexts (117). Integration of genomics with RT decision-making in the clinic, in a retrospective cohort study, yielded a genome-based model for adjusting radiotherapy dose (GARD score) applicable to multiple solid tumors including breast cancer (118). That study was a pioneering attempt towards the individualization of RT dose to tumor radiosensitivity using a genomically guided approach on a large scale.

To advance radiogenomics research, larger cohort studies and multi-center collaborations are necessary and candidate signatures should be validated in independent datasets. Single-cell approaches have the potential to help advance RT because they provide higher resolution with clues to detect intrinsic or induced radioresistance subpopulations. Since radiation is an exogenous intervention affecting gene expression networks, the epigenome is another informative layer for accurate characterization of the underlying mechanisms for radioresistance with the potential to be developed as biomarkers of radioresponse or druggable targets to overcome radioresistance.

The same holistic approach could be applied to mine other clinical features of patients which have been mirrored in imaging and pathology metrics. The clinical images produced by mammography, magnetic resonance imaging (MRI), and ultrasound conceivably embrace many hidden quantitative trends undetectable by humans. Describing an image by its quantitative features, notably using the strengths of sophisticated modeling by AI, provides a chance to combine and mine multi-dimensional information to develop a signature that could be routinely utilized to support clinical decision-making and predict overall survival. This is the aim of the relatively new research field of radiomics, where imaging signatures are extracted manually by predefined features (feature-based radiomics) or identified and generated from the underlying data (deep learning-based radiomics) (119). The essential steps for extracting quantitative image features can be deconstructed into four general tasks: 1- image segmentation or determination of the region of interest; 2- image processing, in order to adjust image features as pixels or intensity to make feature extraction between images with minimum error; 3- feature extraction (calculation of features as a final processing step), per the available guidelines of the Image Biomarker Standardization Initiative (IBSI) that suggest a consensus to report the extracted feature metrics and offer a consensus for standardized feature calculations from all radiomic feature matrices; 4- feature selection/dimension reduction (120). Future studies should examine whether non-coding genomic signatures are associated with medical imaging features by analyzing textural information with AI techniques.

Approximately 70-75% of human lncRNAs are primate-specific (33, 121), hence they provide a promising new line of therapy that will have more direct outcomes with fewer side effects. LncRNAs are new members of regulatory networks, as nodes that have evolved recently during evolution. They have not yet had the time to accrete too many new edges for that evolutionary reason. Accordingly, lncRNAs can be efficiently targeted without devastating downstream effects, given that side effects of drugs are often due to disrupting other components of the same complex and more evolutionarily ancient networks that a drug target is within. The distinctive features of lncRNAs identify them as advantageous targets for personalized medicine in radiation oncology and beyond. LncRNAs often exhibit high levels of variation in expression among individuals than protein-coding genes (122–124). As a result of these profiles, tailored therapies can be developed for each patient, based on their unique lncRNA expression profile (125). Moreover, they demonstrate lower expression levels, yet their expression is more specific to particular diseases, tissues, and cell types when compared to proteins or small RNAs (123, 126). This particularity has sparked an increasing interest in the capabilities of them as candidates for targeted and personalized treatments, offering reduced on-target toxicity to healthy cells and tissues.

However, despite the development of pan-cancer genomic radiosensitivity signatures and of signatures that provide predictions of radiation response in a range of different cancers, the development of assays that incorporate lncRNA signatures is still at the nascent (106). Regardless of the underlying reason, many proposed signatures for identifying intrinsic radiosensitivity show minimal to no overlap in their gene sets. LncRNAs serve as key regulators in numerous signaling pathways and act as central hubs in cellular processes. Therefore, comparing lncRNAs across not only different samples of same tumor type, but also different cancers have the potential to reveal more consistent gene expression profiles. Due to their dysregulation in multiple tumor types by considering their cancer type-specific functions, they may be ideal next-generation targets as tumor-agnostic RNA-based therapeutics (127). Due to the limited therapeutic targets available in breast cancer, obvious limitations of today’s surgical, radiation, and chemotherapy options, and a particular paucity of treatment options in TNBC – where numerous lncRNAs are directly implicated in the pathogenesis (12), lncRNAs as a class of targets merit further in-depth investigation.

## 6 Conclusions and future perspectives

The profound impact of RT in improving the overall survival of breast cancer patients has been frequently evidenced by large-cohort studies (4, 5). However, continual observation of radiation side effects and radioresistance in breast cancer patients implicates that we have not yet arrived at our sought-after destination in radiation oncology: a state where all patients receive their own individualized radiation protocols which hold the maximum benefit and the most advantageous risk-to-benefits ratio. Since genetic background and molecular tumor heterogeneity are among the major contributors to patients’ RT response, in this review - to evaluate where we stand in the field of radiation oncology - we undertook a comprehensive search of peer-reviewed literature referenced in PubMed and Google Scholar, in order to identify studies with a focus on dissecting the molecular basis of radioresistance and radiosensitivity in breast cancer. As we have summarized, the radiation-related pathways modulated by these lncRNAs include but are not limited to DNA damage repair, cell cycle, cancer stem cells phenotype and apoptosis. The development of sophisticated imaging technologies and radio-modifier compounds have positively reshaped RT. However, the advent of big data through the integration of ‘omics approaches will allow the field to more precisely define the most informative predictors.

Immune cells contribute fundamentally to the tumor microenvironment. Due to the challenges related to modeling immunity *in vitro*, immune system-related lncRNAs discovered from cell culture-based studies may not portray the bona fide players in the field. The advent of single-cell and third-generation sequencing is expected to yield additional insights on the subtypes of and the differences in tumor immune infiltration. Hence, more *in vivo* work on radiosensitivity- and radioresistance-associated lncRNAs, including in the tumor microenvironment context, is needed in animal models. However, just as in *in vitro* models, lncRNAs pose challenges in animal models. The fact that the majority of human lncRNA genes have no homologs outside of primates hinders performing endogenous loss of function experiments in rodents. However, humanized mice containing primate-specific lncRNA genes can serve as an alternative. Furthermore, human organoid systems that can replace animal models will ultimately be necessary.

The field of RT, from the discovery of X−rays by Wilhelm Conrad Röntgen in 1895 (128) to the modern era of its unequivocal application in clinics, has witnessed, and fundamentally grown through, the seminal discoveries by grand scientists as Nikola Tesla, Mihajlo Idvorski Pupin, and Maria Sklodowska-Curie on its path (129). The parallel technological breakthroughs in other clinical modules complementary to the field of radiation oncology indicate that we are expecting a treasure trove of data, with the potential to be integrated to eventually prescribe individualized radiation schedules to improve patients’ overall survival. Genomics and radiomics are prominent contributors to this field. Neither Wilhelm Roentgen’s discovery of X-rays, nor Mendel’s foundational determination of the principles of inheritance, could have possibly envisaged the incredible impact of their discoveries in diverse clinical modules and on this type of scale.

LncRNAomics offers promising improvements in cancer treatment. Their high variability in expression among individuals and tissue-specificity render them prime candidates for exploitation in precision medicine. LncRNAs can serve as biomarkers for diagnosis and prognosis, helping identify patients who may benefit from specific radiotherapy protocols. Targeting lncRNAs involved in DNA damage repair can enhance radiotherapy effectiveness. Combining lncRNAomics with imaging and clinical data can lead to more personalized treatment plans. Clinical trials focusing on integrating lncRNAomics and radiation oncology are crucial for developing new, effective treatment protocols. To move towards clinical trials, therapeutic lncRNAs are first selected and validated *in vitro*. As lncRNAs could be therapeutically targeted by antisense oligonucleotides before (ASOs), siRNAs or small molecules this process is followed by identifying the most effective molecules. Subsequent studies then focus on confirming their efficacy and safety in appropriate disease models.

Future research in radiation oncology and lncRNAomics faces challenges and opportunities. Technological advancements like hybrid MRI and PET scans are crucial for precise tumor targeting but are costly and require specialized training. The main challenge in lncRNA research is the limited knowledge derived from a small number of studied lncRNAs, which hinders our grasp of their mechanisms, functions, and structures. Overcoming the hurdle of delivering oligonucleotides to solid tumors remains a critical goal, despite significant progress in the field (125). As a result of refinements in AI and high-level statistical modeling, the implementation of molecular-guided treatment strategies at the resolution of one individual person has emerged as a final and reachable goal of precision medicine. Breast cancer is one of the leading cancers from the viewpoints of incidence, mortality, and cost; therefore, it will enormously benefit from incorporating a functional understanding of the role of lncRNA genes – the majority of human genes and a huge but still poorly understood pool of oncogenes, tumor suppressors, and radioresponse modifiers – into all treatment modules, from diagnosis and prediction response to designing rational therapeutic strategies.
