# Corrigendum: Radiotherapy and breast cancer: finally, an lncRNA perspective on radiosensitivity and radioresistance

**DOI:** 10.3389/fonc.2024.1507032

**Published:** 2024-12-11

**Authors:** Fatemeh Yazarlou, Ivan Martinez, Leonard Lipovich

**Affiliations:** ^1^ Center for Childhood Cancer, Abigail Wexner Research Institute at Nationwide Children’s Hospital, Columbus, OH, United States; ^2^ Department of Microbiology, Immunology and Cell Biology, West Virginia University, Morgantown, WV, United States; ^3^ Department of Biology, College of Science, Mathematics, and Technology, Wenzhou-Kean University, Wenzhou, China; ^4^ Wenzhou Municipal Key Laboratory for Applied Biomedical and Biopharmaceutical Informatics, Wenzhou-Kean University, Wenzhou, China; ^5^ Zhejiang Bioinformatics International Science and Technology Cooperation Center, Wenzhou-Kean University, Wenzhou, China; ^6^ Shenzhen Huayuan Biological Science Research Institute, Shenzhen Huayuan Biotechnology Co. Ltd., Shenzhen, China; ^7^ Center for Molecular Medicine and Genetics, School of Medicine, Wayne State University, Detroit, MI, United States

**Keywords:** breast neoplasms, long non-coding RNAs, non-coding RNAs, precision medicine, radioresistance, radiosensitivity, radiotherapy, genomics

In the published article, there was an error regarding the affiliations for Leonard Lipovich. As well as having affiliations 3, 4, 5, they should also have another two affiliations. The correct affiliation list for Leonard Lipovich after revision appears below:^3^Department of Biology, College of Science, Mathematics, and Technology, Wenzhou-Kean University, Wenzhou, China^4^Wenzhou Municipal Key Laboratory for Applied Biomedical and Biopharmaceutical Informatics, Wenzhou-Kean University, Wenzhou, China^5^Zhejiang Bioinformatics International Science and Technology Cooperation Center, Wenzhou-Kean University, Wenzhou, China^6^Shenzhen Huayuan Biological Science Research Institute, Shenzhen Huayuan Biotechnology Co. Ltd., Shenzhen, China^7^Center for Molecular Medicine and Genetics, School of Medicine, Wayne State University, Detroit, MI, United States

In the published article, there was an omission in the **Funding** statement. Leonard Lipovich, PhD, was funded by the Wenzhou-Kean University faculty start-up research grant, the Wenzhou Municipal Key Laboratory for Applied Biomedical and the Biopharmaceutical Informatics (WB20211227000125), and the Zhejiang Bioinformatics International Science and Technology Cooperation Center at Wenzhou-Kean University (WB20210429000008). The correct **Funding** statement appears below.

